# Unusual Form of Intus-Susceptio of the Colon

**Published:** 1845-07

**Authors:** 


*Unusual form of Intus-Susceptio of the Colon.*—Dr. Harrison said he would briefly detail to the society the particulars of a very unusual case: he had himself never met a similar one, nor had any of the authorities alluded to an exactly parallel occurrence. Some time since, Dr. Gason of Enniskerry, a gentleman extremely well informed in his profession, had sent the patient to him, who was then much emaciated, and with a countenance expressive of great suffering and distress, such as is observed in internal malignant disease. Vomiting was so incessant that the patient could hardly speak. On examination, a tumor, about the size of an orange, was found near the umbilicus, between it and the ribs of the left side; it could be moved up and down, and was free from pain at times, except on pressure. He was much in doubt about the nature of this tumor, but formed a conjecture that it was a malignant growth from the omentum between the colon and stomach. He saw that it could not be an aneurism, and its situation was too low to induce him to suppose the disease was connected with either the liver, spleen or stomach. He would not enter into a detail of the various remedies employed, all of which completely failed to give the slightest relief. The poor man was at times very free from suffering, but at other times he would scream out and say—"Kill me or cut me open!" The only medicine that at all benefitted him was opium, which he continued to take till his death. On examination of the abdomen after death, very little appearance of disease presented itself at first. There was no general inflammation of the peritoneum or of the omentum; but on raising up the latter structure, and examining the colon, an intus-susception of this intestine was seen to have taken place, the descending portion of it being carried up to the transverse arch, probably for an extent of three or four inches. The transverse arch being laid open, Doctor Harrison exhibited the lower portion of intestine lying in the upper, with its orifice resembling an os uteri projecting into a dilated vagina; this contracted appearance of the orifice might, he thought, in some degree, account for the violence of the pain that had been suffered. The intus-suscepted portion of the intestine was found in an ulcerated condition, accounting for the unhealthy discharge that had existed during life. Here was a very remarkable form of intus-susception totally unlike those usually seen. When Hunter, in speaking of the affection, observes that such an occurrence is possible, he talks of the two species of the disease—the one progressive, in which the invagination may go on increasing from above downwards—the other, the retrograde form, the lower portion of the tube being received into the upper, as in the case under consideration, however, he gives no examples of this occurrence. Sir Everard Home mentions one case of retrograde species in the small intestines—a point in which the present case possessed additional interest, for the intus-susception was here situated in the colon, while the small intestines or the cœcum are the parts usually involved. Cruveilhier had never met with this form of the disease, and gives no plate representing the affection. Various opinions respecting the nature of the case were entertained, not one who saw it having diagnosed it, except indeed Dr. Law, who, when he first examined the patient, at once observed that he knew of nothing it resembled so strongly as intus-susception. For himself, he must confess he had not for a moment formed such an opinion. No remedy is known for the disease; the boasted one of the ancients—metallic mercury—being found to be as inefficacious as the rest; purgatives given by the mouth can effect nothing; Hunter says they generally do more harm than good; he concieves that violent vomiting might reverse the peristaltic action, but he (Dr. Harrison) believed very little reliance could be placed on any aid of a mechanical nature. With the exhibition of opium and perfect quiet, it might be hoped that an adhesion would take place between the opposed serous surfaces of the intestine, and the internal cylinder, by this means gradually discharged by the anus; for many cases have been observed in which so many as two or three feet of intestine had come away. The present case, in which no hope of a favorable issue could be at all calculated on, might (had a diagnosis of its true nature been made) have been a good one for the lumbar operation. Had an opening been made in the right lumbar colon, a little above the cœcum, it might reasonably be expected that he would have survived. With regard to the operation of opening the colon in the lumbar region, it appeared to him, judging anatomically, that the right ought to be chosen in preference to the left portion of it, which latter was the one hitherto selected. The right division would be found to be least covered by peritoneum, and to have much less mesocolon, and on making examinations of both portions he had found the right colon generally deficient of peritoneum on its posterior aspect. He thought this case would be interesting to the society for many reasons—the length of time the patient had endured it, upwards of two months, its being an instance of the retrograde species, the impossibility of diagnosing it, and its relation to the very interesting subject lately under discussion.

Dr. O'Beirne begged to trouble the society with one or two observations on the subject of intus-susception, respecting which he had an opportunity of arriving at some facts of which he had as yet said nothing. He had observed (and he wished to know if Dr. H. had made a similar remark in his case,) that in cases of intus-susception, whether progressive or retrogressive, that had come under his observation, the intestine above the intus-suscepted portion was considerably dilated, while the part below it was much contracted, and that the sigmoid flexure of the colon, instead of lying in the iliac region, lay in the pelvis, doubled over the rectum, and to its left side. On passing the tube in these cases, with a view of relieving the bowels, the instrument, instead of passing upwards, followed the course of the sigmoid flexure into the pelvis, and on passing the finger into the rectum, by the side of the tube, a considerable portion of its upper extremity was felt to the left, and with a double of intestine intervening. From this state of the intestine nothing could be gained in cases of intus-susception from the introduction of the tube per anum; but, in recent cases particularly, the most decided advantage must, he thought, result from the introduction of a tube into the stomach, and leaving it there until the flatus distending the upper portion of the intestine, had escaped, and in this way enabling the invaginated portion to liberate itself. He would state, however, that since he had adopted this opinion, he had no opportunity of verifying it by practice. He would conclude by observing that the occurrence of the tube turning back, and passing into the pelvis, is not confined to cases of intus-susception, but also occurs in cases of internal strangulation from newly-formed internal bands. In a case, therefore, where no tumor exists, as in intus-susception, we might fairly diagnose the existence of internal bands, and on referring to some of the early numbers of the *Medical Press*, it would be found that he (Dr. O'B.) confidently foretold their existence in at least two instances.

Dr. H. Kennedy obvserved that having seen this interesting case, it did not appear to him one which at any time called for an operation; and for the simple reason, that at no period was there any positive obstruction of the bowels. They were, indeed, slow, but acted every second, if not every day. The vomiting, too, which was at first obstinate, abated much during his stay in the hospital, so much so, that four, and even five days passed without any. Besides all this, the case lasted nearly three months—a state of things not to be reconciled with obstruction of the bowels. Before sitting down, he wished to mention one hint in the treatment of such cases, and which he believed he had seen in one of the volumes of Guy's Hospital Reports. It consisted in throwing air into the rectum in such qnantitities as to cause the intestine to unfold itself, as it were.—*Dublin Med. Press.*

